# 

**Published:** 1804-05-01

**Authors:** 


### To CORRESPONDENTS.

Communications are received from Dr. Moodie, Mr. Scott, Mr. Stott, Perscrutator, and a Student of Pharmacy.

